# Decision-Making Content of Advance Care Planning in Patients With Heart Failure: A Retrospective Cross-Sectional Study

**DOI:** 10.7759/cureus.91976

**Published:** 2025-09-10

**Authors:** Yuya Tamaki, Hiroaki Sakuma, Ayane Taba, Rinko Morikone, Shuichiro Ikemiya, Koji Yonemoto, Yusuke Ohya, Haruno Nagata

**Affiliations:** 1 Department of Gerontological Nursing, School of Health Sciences, Faculty of Medicine, University of the Ryukyus, Okinawa, JPN; 2 Department of Nursing, University of the Ryukyus Hospital, Okinawa, JPN; 3 Department of Rehabilitation Medicine, University of the Ryukyus Hospital, Okinawa, JPN; 4 Department of Biostatistics, School of Health Sciences, Faculty of Medicine, University of the Ryukyus, Okinawa, JPN; 5 Department of Medicine, University of the Ryukyus Hospital, Okinawa, JPN; 6 Department of Cardiovascular Medicine, Nephrology, and Neurology, University of the Ryukyus Hospital, Okinawa, JPN

**Keywords:** advance care planning, characteristics, decision-making, heart failure, japan

## Abstract

Introduction

Advance care planning (ACP) is a process in which individuals collaboratively discuss and share their values and wishes regarding future medical care. Domestic and international heart failure (HF) guidelines recommend early ACP for patients after HF diagnosis. Various issues remain unresolved, including insufficient preparedness on the part of patients and differences in perceptions of ACP among healthcare providers. Therefore, we aimed to examine the current status and issues of ACP by organizing the characteristics of the ACP decision-making content in patients with HF.

Methods

The patient characteristics and implementation of ACP were retrospectively investigated from the electronic medical records of patients with HF who had been admitted to a single university hospital and had undergone ACP. To examine the characteristics of recognition of HF stage and decision-making content according to differences in HF severity, patients were classified into stage C and stage D based on the American College of Cardiology Foundation/American Heart Association (ACCF/AHA) HF staging system. Furthermore, within each stage, patients were categorized according to age, sex, and number of hospitalizations for HF, and the ACP decision-making content was summarized using descriptive statistics.

Results

The study included 177 patients with HF (131 with stage C; 46 with stage D). There were discrepancies in perceptions of ACCF/AHA HF stage among older patients with HF; only 19 (39.6%) of patients with HF stage C correctly identified their stage, and only five (21.7%) of those with stage D did so. Despite demographic differences, 12 (85.7%) of respondents were aware of the existence of a surrogate decision-maker; however, fewer than half had engaged in ACP-related discussions with a surrogate decision-maker. In addition, patients with HF stage D aged ≥65 years, patients who were male, and patients with a history of two or more hospitalizations for HF were each found to have more than half of individuals expressing a preference to spend the end-of-life period at home; however, fewer than half in any group had engaged in discussions about ACP with their surrogate decision-makers. Regarding impressions after ACP, 29 (87.9%) of those aged <65 years with stage C responded “good.”

Conclusions

Older patients with HF had a gap in their perception of their HF stage. Despite Japan’s family-centered decision-making culture, the current ACP implementation rate, including that of family members, is low. Therefore, medical staff should play a coordinating role in promoting ACP.

## Introduction

At the end-of-life stage, approximately 70% of patients lose the ability to make decisions, highlighting the importance of confirming a patient's wishes in advance [1]. Advance care planning (ACP) has been recommended as a method to ensure these decisions are made beforehand [2]. Although the definition of ACP has changed over time, an international definition from 2017 states that individuals with decision-making capacity should clarify and consider the implications and consequences of serious illness scenarios and discuss goals and preferences regarding future medical care with family members and healthcare providers [3]. It further recommends that any wishes be documented and reviewed regularly and that a surrogate decision-maker be identified so that if the individual is unable to make decisions at some point, their wishes can be considered [3].

Predicting the prognosis of patients with heart failure (HF) is difficult due to the gradual deterioration of the disease with repeated exacerbations and remissions and the possibility of sudden death due to rapid changes in the disease state. Therefore, domestic and international HF guidelines recommend that ACP be performed early in patients after HF diagnosis [4-7]. Topics discussed in ACP include ACP in oncology [8], the recommended elements of international ACP [3], and HF guidelines [4, 9]. Regarding the actual situation of ACP in patients with HF, a survey study of 171 patients with HF from a single institution in Japan between 2017 and 2020 reported on end-of-life care and factors considered important for ACP. Of all the participants, only 21.7% had actually undergone ACP; however, 74.3% considered it important [10]. Internationally, there have been reports on the status of advance directives for patients with HF [11]. Decision-making in ACP is influenced by various factors, such as the impact of aging on decision-making capacity [12], sex-related differences in neural structures involved in emotion and decision-making [13], and variation in the willingness to engage in ACP according to the number of prior HF hospitalizations [10]. However, the characteristics of decision-making content according to patient attributes have not been sufficiently investigated. One likely reason is the generally low implementation rate of ACP both domestically and internationally, which has limited the ability to conduct sufficiently powered analyses. For example, the reported ACP implementation rate among HF patients in Japan is 11.1% [14], while a study from a United States university hospital reported a rate of 15.6% [15]. Furthermore, it has been reported that confirming patients’ awareness of their own disease status is an essential preparatory step for ACP decision-making [16], as the content of decisions may change according to disease severity. Therefore, clarifying how disease perception and decision-making content differ according to patient attributes and the severity of HF could inform healthcare providers’ approaches to decision-making support. In this study, we aimed to identify the characteristics of ACP decision-making content among HF patients with diverse attributes, classified by HF stage, in order to explore challenges and considerations in supporting ACP decision-making.

## Materials and methods

Study design and participants

This was a retrospective cross-sectional study based on electronic medical records. The study included all adult patients with the American College of Cardiology Foundation/American Heart Association (ACCF/AHA) stage C and stage D HF who participated in cardiac rehabilitation at the University of the Ryukyus Hospital and underwent ACP between December 2016 and July 2023. At this hospital, ACP is performed in patients at a high risk of HF rehospitalization and in those requiring adjustments to their care environment. In this study, patients at a high risk of HF rehospitalization were defined as those who were anticipated to have difficulty managing precipitating factors for HF exacerbation or responding appropriately when symptoms appeared. Patients requiring adjustments to their care environment were defined as those who needed support with HF self-care management and activities of daily living but had insufficient assistance from family members or others. Determination of whether patients met these definitions was made through discussion between the attending physician and the clinician responsible for conducting ACP. The exclusion criteria were patients who had difficulty communicating due to severe dementia or aphasia, or when the patient declined ACP. The criterion for severe dementia was defined as a documented history in the medical record of a diagnosis corresponding to the International Statistical Classification of Diseases and Related Health Problems, 10th Revision (ICD‑10) codes F00-F03, along with impairment in all of the following aspects of orientation: understanding of one’s own age and date of birth, recognition of surrounding persons, awareness of place, and comprehension of the meaning of date and time, as determined by the investigators.

Although ACP encompasses various elements, in the present study, we included those that could be retrospectively investigated: recognition of the ACCF/AHA HF stage, preferences regarding end-of-life care and treatment methods, presence and experience of discussions with surrogate decision-makers, and impressions after ACP. A dedicated format was created within electronic medical records to record the ACP decision-making. Because ACP is performed repeatedly, patients who underwent their first ACP at our hospital during the study period were included.

Variables

All study data were extracted from electronic medical records. The basic attributes used in this study were age, sex, presence of a cohabitant, occupational status, social service utilization, and smoking history. The following clinical findings were used: causative disease of HF, HF history, and medical history (the presence of mental disorders was defined as a documented history in the medical records of a diagnosis corresponding to ICD-10 codes F1-F8, and dementia was defined as a documented history of a diagnosis corresponding to ICD-10 codes F00-F03). HF-related clinical findings included number of years since HF diagnosis, number of HF hospitalizations, classification of HF by the New York Heart Association (NYHA) on the date of ACP, the Seattle Heart Failure Model (SHFM) [17], ACCF/AHA HF stage classification, left ventricular ejection fraction, and body mass index on or immediately before the date of ACP. Blood test data included N-terminal pro-B-type natriuretic peptide, hemoglobin, albumin, and estimated glomerular filtration rate on or immediately before the date of ACP. ACP implementation status included the types of specialist qualifications for ACP facilitators, number of days between admission and ACP, and activities of daily living level (Barthel index) [18] on the ACP date. The following ACP content was used: awareness of the ACCF/AHA HF stage classification, decision-making for end-of-life care location and regarding end-of-life treatments, experience with surrogate decision-makers and ACP-related discussions, and impressions after ACP.

Data analysis

Descriptive statistics were used for all variables. Continuous variables were expressed as mean and standard deviation or as median and interquartile range, depending on the characteristics of the distribution. Nominal variables were expressed as n (%). ACP implementation was represented by ACCF/AHA HF stages C and D and categorized by age (<65 and ≥65 years), sex, and number of HF hospitalizations. In addition, because this study presented the results using only descriptive statistics, no special imputation or exclusion was performed for missing data, and the analysis was conducted and reported within the range of the available data. Statistical analysis was performed using JMP version 17.2 (SAS Institute Inc., Cary, NC, USA).

Ethics approval

This study was approved by the Ethics Committee for Clinical Research of University of the Ryukyus (approval number: 23-2127-01-00-00). This was a retrospective study, and an opt-out method was used. The study was disclosed on the website of the University of the Ryukyus Hospital, and an opportunity to refuse to participate in the study was provided.

## Results

Characteristics of participants

A total of 177 patients with HF underwent ACP between December 2016 and July 2023. Of these, 131 were stage C, and 46 were stage D. All patients consented to ACP during the same period. Patient characteristics for stages C and D are presented in Table 1. In stage C, the median age was 74 years, and 22 (16.8%) of the patients lived alone. Valvular disease was the most common underlying HF, and a history of cardiac surgery was the most common history of HF. The median number of HF hospitalizations was one, and the median SHFM-estimated one-year survival rate was 87.0%. Of the ACP facilitators, 114 (87%) were nurses. The median length of time from admission to ACP was 12 days. In stage D, the median age was 73 years, four (8.7%) lived alone, and 15 (32.6%) used social services. Cardiomyopathy was the most common underlying HF disease, and a history of cardiac surgery and ventricular tachycardia or fibrillation was the most common finding. The median number of HF hospitalizations was 2.5, and the median SHFM-estimated one-year survival rate was 76.4%. Of ACP facilitators, 38 (82.6%) were nurses. The median length of time from admission to ACP was 18.5 days.

**Table 1:** Characteristics of participants according to ACCF/AHA HF stage classification ACCF/AHA, American College of Cardiology Foundation/American Heart Association; BI, Barthel index; BMI, body mass index; eGFR, estimated glomerular filtration rate; HF, heart failure; IQR, interquartile range; LVEF, left ventricular ejection fraction; NT-pro BNP, N-terminal pro–B-type natriuretic peptide; NYHA, New York Heart Association; OT, occupational therapist; PT, physical therapist; RN, registered nurse; SD, standard deviation; SHFM, Seattle Heart Failure Model; VF, ventricular fibrillation; VT, ventricular tachycardia

Variables	Stage C (N = 131)	Stage D (N = 46)
Demographic data		
Age, median (IQR)	74.0 (63.0-85.0)	73.0 (58.5-86.0)
Women, n (%)	51 (38.9)	18 (39.1)
Living alone, n (%)	22 (16.8)	4 (8.7)
Occupation, n (%)	36 (27.5)	9 (19.6)
Utilization of social services, n (%)	22 (16.8)	15 (32.6)
History of smoking (none), n (%)	66 (50.4)	24 (52.2)
History of smoking (past), n (%)	54 (41.2)	22 (47.8)
History of smoking (current), n (%)	11 (8.4)	0 (0.0)
Etiology of HF		
Ischemic, n (%)	29 (22.1)	15 (32.6)
Valvular, n (%)	44 (33.6)	14 (30.4)
Hypertension, n (%)	13 (9.9)	0 (0.0)
Cardiomyopathy, n (%)	33 (25.2)	16 (34.8)
Others, n (%)	50 (38.2)	10 (21.7)
Medical history		
Cardiac surgery, n (%)	44 (33.6)	16 (34.8)
VT/VF, n (%)	10 (7.6)	16 (34.8)
Dialysis, n (%)	7 (5.3)	1 (2.2)
Stroke, n (%)	18 (13.7)	3 (6.5)
Mental illness, n (%)	8 (6.1)	3 (6.5)
Dementia, n (%)	5 (3.8)	3 (6.5)
HF-related clinical findings		
Years since diagnosis of HF, median (IQR)	1.0 (1.0-3.0)	2.5 (1.0-5.5)
Number of HF hospitalizations, median (IQR)	1.0 (1.0-2.0)	2.0 (1.0-3.0)
NYHA Ⅰ, n (%)	13 (9.9)	0 (0.0)
NYHA Ⅱ, n (%)	90 (68.7)	0 (0.0)
NYHA Ⅲ, n (%)	27 (20.6)	41 (89.1)
NYHA Ⅳ, n (%)	1 (0.8)	5 (10.9)
SHFM-estimated 1 year survival rate, median (IQR)	87.0 (76.5-91.7)	76.4 (63.5-82.3)
LVEF, median (IQR)	44.7 (26.7-60.7)	25.7 (18.6-47.0)
BMI, mean ± SD	23.6 ± 5.2	22.0 ± 4.6
Blood test data		
NT-pro BNP, median (IQR)	3,256 (1,317-7,331)	6,620 (2,707-14,129)
Hemoglobin, mean ± SD	11.9 ± 2.3	11.4 ± 2.2
Albumin, mean ± SD	3.3 ± 0.6	3.2 ± 0.6
eGFR, mean ± SD	43.6 ± 23.6	45.2 ± 24.0
ACP implementation status		
Specialist qualifications for ACP facilitators RN, n (%)	114 (87.0)	38 (82.6)
Specialist qualifications for ACP facilitators PT, n (%)	5 (3.8)	1 (2.2)
Specialist qualifications for ACP facilitators OT, n (%)	12 (9.2)	7 (15.2)
Days from admission to ACP, median (IQR)	12.0 (8.0-20.0)	18.5 (9.8-28.3)
BI on the day of ACP implementation, median (IQR)	90.0 (75.0-100.0)	75.0 (48.8-90.0)

ACP by characteristics

For each stage, the decision-making content of ACP was categorized by age (<65 and ≥65 years) (Table 2). Among stage C respondents, 15 (65.2%) of those aged <65 years and 19 (39.6%) of those aged ≥65 years correctly identified their ACCF/AHA HF stage. In addition, 12 (25.0%) of those aged ≥65 years reported that they did not know their stage. Of the respondents aged ≥65 years, 16 (17.6%) were unsure of their preferred location for end-of-life care. At least 33 (91.7%) of respondents, regardless of age, reported having a surrogate decision-maker; however, eight (25.8%) had actually discussed ACP with the surrogate decision-maker. As for their impressions after ACP, 29 (87.9%) of those aged <65 years and 56 (68.3%) of those age ≥65 years answered that the ACP was “good.” Among stage D respondents, eight (72.7%) of those aged <65 years and five (21.7%) of those aged ≥65 years correctly identified their ACCF/AHA HF stage. In addition, nine (39.1%) of those aged ≥65 years were unsure about their disease stage. Regardless of age, 14 (87.5%) reported having a surrogate decision-maker; however, five (38.5%) of these respondents had actually conducted ACP discussions with the surrogate decision-maker.

**Table 2:** ACP decision-making content stratified by age across ACCF/AHA HF stages C and D ACCF/AHA, American College of Cardiology Foundation/American Heart Association; ACP, advance care planning; HF, heart failure

ACP Decision-Making Elements	Stage C (N = 131), Age < 65 (N = 36)	Stage C (N = 131), Age ≥ 65 (N = 95)	Stage D (N = 46), Age < 65 (N = 16)	Stage D (N = 46), Age ≥ 65 (N = 30)
					Awareness of ACCF/AHA HF stage classification				
Correctly identified HF stage, n (%)	15 (65.2)	19 (39.6)	8 (72.7)	5 (21.7)
Underestimation of HF stage, n (%)	2 (8.7)	13 (27.1)	2 (18.2)	9 (39.1)
Overestimation of HF stage, n (%)	2 (8.7)	4 (8.3)	0 (0.0)	0 (0.0)
Do not know, n (%)	4 (17.4)	12 (25.0)	1 (9.1)	9 (39.1)
Decision-making for end-of-life care location				
At-home, n (%)	13 (40.6)	43 (47.3)	7 (43.8)	16 (55.2)
Hospital or nursing home, n (%)	13 (40.6)	32 (35.2)	6 (37.5)	11 (37.9)
Residential hospice, n (%)	2 (6.3)	0 (0.0)	1 (6.3)	0 (0.0)
Do not know, n (%)	4 (12.5)	16 (17.6)	2 (12.5)	2 (6.9)
Decision-making regarding end-of-life treatments				
All treatments, including invasive treatments, n (%)	23 (67.7)	42 (46.7)	11 (73.3)	4 (14.3)
Non-invasive medical therapy, n (%)	0 (0.0)	16 (17.8)	1 (6.7)	15 (53.6)
Best supportive care, n (%)	8 (23.5)	16 (17.8)	2 (13.3)	6 (21.4)
Do not know, n (%)	3 (8.8)	16 (17.8)	1 (6.7)	3 (10.7)
Presence of a surrogate decision-maker				
Yes, n (%)	33 (91.7)	83 (95.4)	14 (87.5)	29 (96.7)
No, n (%)	3 (8.3)	3 (3.5)	1 (6.3)	1 (3.3)
Do not know, n (%)	0 (0.0)	1 (1.2)	1 (6.3)	0 (0.0)
ACP experience with selected surrogate decision-makers				
Yes, n (%)	8 (25.8)	21 (26.6)	5 (38.5)	11 (42.3)
No, n (%)	23 (74.2)	58 (73.4)	8 (61.5)	15 (57.7)
Impressions after ACP				
Good, n (%)	29 (87.9)	56 (68.3)	10 (71.4)	19 (70.4)
Not good, n (%)	3 (9.1)	19 (23.2)	3 (21.4)	6 (22.2)
Do not know, n (%)	1 (3.0)	7 (8.5)	1 (7.1)	2 (7.4)

Table 3 shows the decision-making content of ACP for men and women by stage. Of the stage C respondents, 19 (63.3%) of women and 15 (36.6%) of men correctly identified their ACCF/AHA HF stage. Regardless of sex, 25 (89.3%) of the respondents answered that they had a surrogate decision-maker; however, nine (37.5%) had actually discussed ACP with the surrogate decision-maker. Of the stage D respondents, none of the women responded “do not know” regarding their preference for end-of-life care. Regardless of sex, 25 (89.3%) of the respondents answered that they had a surrogate decision-maker; however, nine (37.5%) had actually discussed ACP with the surrogate decision-maker. 13 (81.3%) of women and 16 (64.0%) of men answered that they were “satisfied” after ACP was implemented.

**Table 3:** ACP decision-making content stratified by sex across ACCF/AHA HF stages C and D ACCF/AHA, American College of Cardiology Foundation/American Heart Association; ACP, advance care planning; HF, heart failure

ACP Decision-Making Elements	Stage C (N = 131), Women (N = 51)	Stage C (N = 131), Men (N = 80)	Stage D (N = 46), Women (N = 18)	Stage D (N = 46), Men (N = 28)
					Awareness of ACCF/AHA HF stage classification				
Correctly identified HF stage, n (%)	19 (63.3)	15 (36.6)	5 (35.7)	8 (40.0)
Underestimation of HF stage, n (%)	5 (16.7)	10 (24.4)	6 (42.9)	5 (25.0)
Overestimation of HF stage, n (%)	1 (3.3)	5 (12.2)	0 (0.0)	0 (0.0)
Do not know, n (%)	5 (16.7)	11 (26.8)	3 (21.4)	7 (35.0)
Decision-making for end-of-life care location				
At-home, n (%)	20 (40.8)	36 (48.7)	8 (44.4)	15 (55.6)
Hospital or nursing home, n (%)	21 (42.9)	24 (32.4)	9 (50.0)	8 (29.6)
Residential hospice, n (%)	2 (4.1)	0 (0.0)	1 (5.6)	0 (0.0)
Do not know, n (%)	6 (12.2)	14 (18.9)	0 (0.0)	4 (14.8)
Decision-making regarding end-of-life treatments				
All treatments, including invasive treatments, n (%)	25 (52.1)	40 (52.6)	4 (25.0)	11 (40.7)
Non-invasive medical therapy, n (%)	9 (18.8)	7 (9.2)	7 (43.8)	9 (33.3)
Best supportive care, n (%)	5 (10.4)	19 (25.0)	3 (18.8)	5 (18.5)
Do not know, n (%)	9 (18.8)	10 (13.2)	2 (12.5)	2 (7.4)
Presence of a surrogate decision-maker				
Yes, n (%)	47 (95.9)	69 (93.2)	18 (100.0)	25 (89.3)
No, n (%)	2 (4.1)	4 (5.4)	0 (0.0)	2 (7.1)
Do not know, n (%)	0 (0.0)	1 (1.4)	0 (0.0)	1 (3.6)
ACP experience with selected surrogate decision-makers				
Yes, n (%)	11 (25.0)	18 (27.3)	7 (46.7)	9 (37.5)
No, n (%)	33 (75.0)	48 (72.7)	8 (53.3)	15 (62.5)
Impressions after ACP				
Good, n (%)	36 (78.3)	49 (71.0)	13 (81.3)	16 (64.0)
Not good, n (%)	7 (15.2)	15 (21.7)	1 (6.3)	8 (32.0)
Do not know, n (%)	3 (6.5)	5 (7.3)	2 (12.5)	1 (4.0)

Table 4 shows the ACP decision-making by stage classified by first and second or later hospitalizations. Of the stage C respondents, 19 (45.2%) correctly identified their ACCF/AHA HF stage at the time of their first HF admission and 15 (51.7%) at the time of their second and subsequent admissions. Regardless of the number of HF hospitalizations, 65 (94.2%) of the respondents reported having a surrogate decision-maker; however, 15 (23.4%) had actually discussed ACP with the surrogate decision-maker. Of the stage D respondents, as for the perception of the ACCF/AHA HF stage of participants, four (33.3%) of the respondents in the first HF hospitalization had the same perception as the actual stage, and five (41.7%) answered “do not know.” In the second or later HF hospitalizations, two (6.3%) of the respondents answered “do not know” in their preference for location of end-of-life care, and two (6.9%) answered “do not know” in their preference for end-of-life medical care. Regardless of the number of HF hospitalizations, 12 (85.7%) of the respondents reported having a surrogate decision-maker; however, four (33.3%) had actually discussed ACP with that surrogate decision-maker.

**Table 4:** ACP decision-making content stratified by number of HF hospitalizations across ACCF/AHA HF stages C and D ACCF/AHA, American College of Cardiology Foundation/American Heart Association; ACP, advance care planning; HF, heart failure

ACP Decision-Making Elements	Stage C (N = 131), First Hospitalization (N = 74)	Stage C (N = 131), Second or More Hospitalizations (N = 57)	Stage D (N = 46), First Hospitalization (N = 14)	Stage D (N = 46), Second or More Hospitalizations (N = 32)
					Awareness of ACCF/AHA HF stage classification				
Correctly identified HF stage, n (%)	19 (45.2)	15 (51.7)	4 (33.3)	9 (40.9)
Underestimation of HF stage, n (%)	7 (16.7)	8 (27.6)	3 (25.0)	8 (36.4)
Overestimation of HF stage, n (%)	4 (9.5)	2 (6.9)	0 (0.0)	0 (0.0)
Do not know, n (%)	12 (28.6)	4 (13.8)	5 (41.7)	5 (22.7)
Decision-making for end-of-life care location				
At-home, n (%)	33 (47.1)	23 (43.4)	5 (38.5)	18 (56.3)
Hospital or nursing home, n (%)	25 (35.7)	20 (37.7)	6 (46.2)	11 (34.4)
Residential hospice, n (%)	0 (0.0)	2 (3.8)	0 (0.0)	1 (3.1)
Do not know, n (%)	12 (17.1)	8 (15.1)	2 (15.4)	2 (6.3)
Decision-making regarding end-of-life treatments				
All treatments, including invasive treatments, n (%)	33 (46.5)	32 (60.4)	4 (28.6)	11 (37.9)
Non-invasive medical therapy, n (%)	10 (14.1)	6 (11.3)	7 (50.0)	9 (31.0)
Best supportive care, n (%)	15 (21.1)	9 (17.0)	1 (7.1)	7 (24.1)
Do not know, n (%)	13 (18.3)	6 (11.3)	2 (14.3)	2 (6.9)
Presence of a surrogate decision-maker				
Yes, n (%)	65 (94.2)	51 (94.4)	12 (85.7)	31 (96.9)
No, n (%)	3 (4.4)	3 (5.6)	1 (7.1)	1 (3.1)
Do not know, n (%)	1 (1.5)	0 (0.0)	1 (7.1)	0 (0.0)
ACP experience with selected surrogate decision-makers				
Yes, n (%)	15 (23.4)	14 (30.4)	4 (33.3)	12 (44.4)
No, n (%)	49 (76.6)	32 (69.6)	8 (66.7)	15 (55.6)
Impressions after ACP				
Good, n (%)	49 (73.1)	36 (75.0)	10 (71.4)	19 (70.4)
Not good, n (%)	15 (22.4)	7 (14.6)	2 (14.3)	7 (25.9)
Do not know, n (%)	3 (4.5)	5 (10.4)	2 (14.3)	1 (3.7)

**Figure 1:**
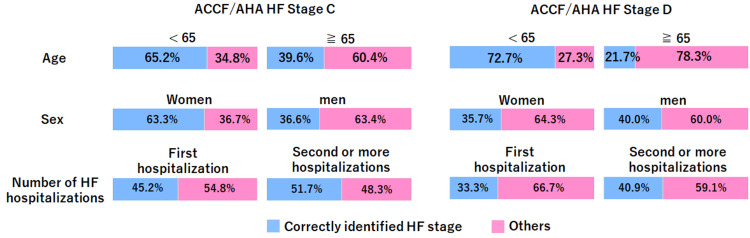
Awareness of ACCF/AHA HF stage classification by different attributes The blue graph represents the proportion of patients who correctly recognized their ACCF/AHA HF stage, while the pink graph shows the proportion of those who either underestimated, overestimated, or responded with “do not know” regarding their HF stage. ACCF/AHA, American College of Cardiology Foundation/American Heart Association; HF, heart failure

Figure 1 presents the awareness of the ACCF/AHA HF stage classification according to various attributes in a graphical form. Particularly among patients with ACCF/AHA HF stage D, a marked difference was observed between age groups: eight (72.7%) aged <65 years correctly recognized their stage, compared with only five (21.7%) aged ≥65 years.

**Figure 2:**
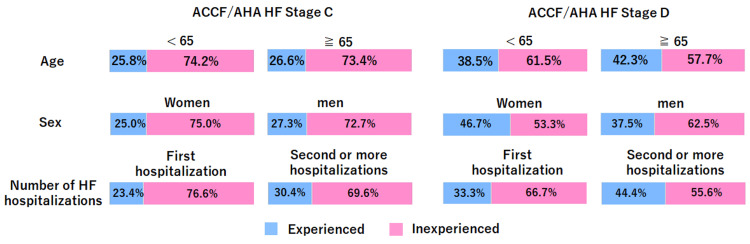
Advance care planning experience with selected surrogate decision-makers by different attributes The blue graph represents respondents who reported having a surrogate decision-maker and had experience engaging in discussions related to advance care planning with their surrogate decision-maker, whereas the pink graph represents those without such experience. ACCF/AHA, American College of Cardiology Foundation/American Heart Association; HF, heart failure

Figure 2 presents the proportion of individuals with a designated surrogate decision-maker who reported having actual experience with ACP discussions according to various attributes in a graphical form. Regardless of demographic attributes, patients, particularly those at stage C, tended to have limited experience with ACP discussions involving a surrogate decision-maker.

**Figure 3:**
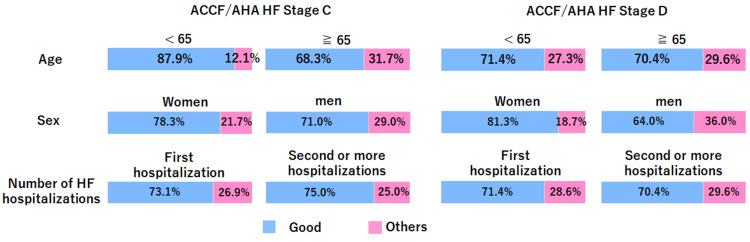
Impressions after advance care planning by different attributes The blue graph represents the proportion of respondents who answered “good” regarding their impressions after the implementation of advance care planning, whereas the pink bar labeled “others” represents the proportion of respondents who answered “not good” or “do not know.” ACCF/AHA, American College of Cardiology Foundation/American Heart Association; HF, heart failure

Figure 3 presents the impressions after ACP implementation according to various attributes in a graphical form. In particular, at stage C, negative experiences tended to be more frequent in those aged ≥65 years, 26 (31.7%), than in those aged <65 years, four (12.1%). On the other hand, the proportion of respondents who reported a negative experience was comparable between the two age groups: three (21.4%) in those aged <65 years and six (22.2%) in those aged ≥65 years.

## Discussion

In this retrospective study, we investigated the challenges involved in supporting ACP decision-making among patients with HF. The decision-making content for each ACP component was not extremely biased by attribute differences, which supports the importance of individualized ACP, as recommended by the International Consensus on ACP [3] and HF guidelines [5]. The main challenges of ACP for patients with HF were as follows: problem 1, older patients with HF have a gap in the perception of disease stage and difficulties in decision making; problem 2, many have a surrogate decision-maker; however, the rate of ACP implementation including the surrogate decision-maker is low; and problem 3, although there was a lot of positive feedback on ACP from participants, there was also negative feedback.

Regarding problem 1, understanding HF is a prerequisite for performing ACP. Among older patients with HF, 19 (39.6%) correctly identified stage C and five (21.7%) correctly identified stage D, indicating a gap in perception. Additionally, regardless of age, sex, or number of hospitalizations, the proportion of patients who underestimated their HF stage increased as the stage advanced. It is conceivable that the severity of cognitive impairment may influence the understanding of HF stage; however, in the present study, patients with severe dementia were excluded. Moreover, even among the included patients, it was difficult to categorize the severity of cognitive impairment, and thus the impact on stage comprehension could not be examined. However, as patients with HF have been reported to have a high prevalence of cognitive dysfunction [19], it is assumed that a considerable proportion of the participants in this study also had impaired cognitive function. Moreover, cognitive function in patients with HF has been reported to be associated with the severity of HF [20], and the difficulty in predicting prognosis in HF often leads patients to overestimate their life expectancy [21]. These factors may contribute to the increased underestimation of HF stage as the disease progresses. In addition, as a cultural characteristic in Japan, it is possible that denial related to the reluctance to think about death and the unwillingness to accept disease progression also plays a role [22] . Therefore, when assessing patients’ awareness of their condition, it is important to consider multiple contributing factors, including whether the lack of awareness is due to insufficient knowledge, impaired comprehension, or a conscious unwillingness to accept the reality of disease progression. In addition, as an educational approach by healthcare professionals, current methods using HF handbooks may not be sufficient to increase the understanding and awareness of the disease stage. It has been reported that ACP using support tools such as videos improves HF knowledge and reduces decision-making difficulties [23]. Further investigation of these associations is warranted.

Regarding problem 2, 12 (85.7%) of the respondents were aware of the existence of a surrogate decision-maker despite differences in demographics; however, less than a majority had actually conducted discussions about ACP with a surrogate decision-maker. In previous international reports, some studies have described the proportion of patients who designated a surrogate decision-maker as part of ACP [24]; however, we found no studies that reported the implementation rate of ACP including surrogate decision-makers. Nevertheless, the low implementation rate of ACP is a common issue not only in Japan but also worldwide. For example, the ACP implementation rate at a university hospital in the United States was reported to be 15.6% [15]. In addition, a study using a database of approximately five million patients in primary care in the United Kingdom reported a striking disparity: 48% of patients with cancer were enrolled in palliative care, whereas only 7% of patients with HF were enrolled, highlighting the challenge of low ACP implementation [25]. Therefore, it can be assumed that the implementation rate of ACP involving surrogate decision-makers is similarly low. In particular, owing to their high-context communication culture and family-centered decision-making culture [26], Japanese people are likely to perceive that their intentions are understood even if they do not express them in words. Therefore, to promote ACP that includes Japanese surrogate decision-makers, medical professionals may need to facilitate the process.

Regarding problem 3, among patients with stage C HF, 29 (87.9%) of those aged <65 years reported that they were glad to have ACP, and 49 (73.1%) of those in their first HF hospitalization reported that they were glad to have ACP, indicating a favorable attitude toward ACP. In a survey of patients with HF at a single center in Japan, although only 21.7% had actually undergone ACP, 74.3% perceived ACP as important [10]. This will encourage the implementation of ACP early after HF diagnosis, as recommended by the HF Guidelines [5]. However, some patients did not perceive ACP as good. Among patients with ACCF/AHA stage D HF, although there were age-related differences in the recognition of disease stage, the proportion of those who reported a negative impression after the implementation of ACP was almost identical between age groups. To our knowledge, we could not identify studies that have directly compared negative attitudes toward ACP across different age groups. However, among patients younger than 65 years, the higher likelihood of correctly recognizing the severity of HF may have led ACP to serve as an opportunity to confront the reality of their condition, which may have resulted in a certain proportion expressing negative impressions. In contrast, in patients aged ≥65 years, insufficient recognition of disease severity may have caused discrepancies in the perceived necessity of ACP between patients and healthcare providers, thereby contributing to negative impressions. Furthermore, given that Japanese individuals are generally resistant to discussing death [22], conversations regarding end-of-life care may also have contributed to unfavorable perceptions. These factors warrant future qualitative investigation; however, it should be acknowledged that a proportion of patients inevitably hold negative views toward ACP. Therefore, decision-making support that is tailored to individual preferences remains essential.

A limitation of this study was that it was a report on ACP conducted at a single university hospital in Japan, and that it included only patients with HF who had undergone ACP; therefore, the findings cannot be generalized to the entire Japanese population. However, there have been no reports that classify the content of ACP decision-making in patients with HF based on patient characteristics and examine the associated challenges; therefore, we consider that this could constitute important insights for the future development of ACP. In this study, the classification of ACCF/AHA HF Stage D was based on the Japanese HF guidelines [4]. However, there is no absolute standard criterion for stage D, and its assessment may vary depending on the clinician’s interpretation. Moreover, because this was a retrospective study, the reliability of the classification cannot be fully assured. Nevertheless, as our findings bear similarity to those reported in a previous study of patients with HF requiring evaluation for potential heart transplantation, as assessed by the SHFM score [27], we consider the likelihood of substantial misclassification to be low. In addition, this was a retrospective study, and the method of selecting the participants and the method of conducting ACP have been modified as ACP has evolved. Therefore, it is not known whether the decision-making content of ACP can be implemented in an appropriate manner and timing. However, the results appear to be close to the best practices, as the guidelines were modified to allow ACPs to be implemented in accordance with the best practices at the time. In this study, we examined the content of ACP decisions implemented by nurses, physical therapists, and occupational therapists. Although physical therapists and occupational therapists were not the primary focus, previous studies have reported that physicians and nurses differ in their perceptions of ACP for patients with HF [28]. Therefore, it is necessary to consider the influence of professional discipline on ACP decision content. However, since the implementation rate by physical therapists and occupational therapists was low (14.1%, n=25), it was difficult to adequately examine this influence in the present study. Further research is required to elucidate the impact of professional discipline on ACP decision content. In addition, ACP in this study was primarily the decision-making content conducted by nurses. ACPs assisted by trained healthcare providers, including nurses, have been reported to be helpful in improving patient and family satisfaction and reducing stress, anxiety, and depression among bereaved families [29]. Nurses seem to be the preferred ACP providers because they are more accessible to patients and families and better positioned to build trusting relationships.

## Conclusions

The content of decision-making for each ACP component was not extremely biased by differences in attributes, reinforcing the importance of individualized ACP. Because older patients with HF have a gap in their perception of the HF stage and the current low ACP implementation rate, including by family members, it is necessary for healthcare providers, including nurses, to serve as coordinators and promote ACP. Addressing the challenges to ACP identified in this study may contribute to the development of strategies tailored to the characteristics of the Japanese population and to the promotion of ACP implementation from an early stage. In the future, to understand the actual status of ACP in Japan as a whole, studies should be conducted in more regions.
